# Voyages in Vacation.—XV

**Published:** 1889-01-19

**Authors:** 


### VOYAGES IN VACATION.—XV. (Continued from page 233.)

### RUSSIAN DINNERS, HOTELS, RESTAURANTS AND AMUSEMENTS.

A CIRCUS was performing during our visit to Moscow, and we went to it, hoping to see the Russian people at their ease. It turned out to be a remarkable performance in many respects. First it was evident that the showman's proclivities, of which Mr. Augustus Druriolanus is a master, had not yet penetrated these regions. For example, the stage properties were conspicuous by their absence, the fittings and decorations were of the most meagre description, and the stud might have been drawn from a regiment of Cossacks. The performance too was singularly simple, unexciting, and uninteresting; there did not appear to be one member of the company, male or female, who could do any of the ordinary circus tricks with even reasonable facility, and no village troupe which we have seen was anything like so feeble or ill found in every respect. There was, however, a good audience, and the people seemed to be well satisfied with the programme provided for them. No doubt the position of Moscow and the condition of its population are not such as to attract speculators in public entertinments, although the circus is located in a permanent building, and is generally open we understand. On the other hand, Moscow possesses a very fine opera house, centrally situated, and sumptuously furnished and found in all respects; it is filled by immense audiences during the season, and Russian opera frequently commends itself to strangers, as it presents many characteristic features which they would not be likely to meet with elsewhere. While on the subject of amusements, it may be incidentally stated that the Swedes are very fond of circuses, Stockholm supporting at least two, which occupy fine buildings of considerable extent. The performances witnessed in Stockholm at the circuses are equal to any in the world, a pretty good proof that this kind of talent is either excluded from, or at any-rate not attracted to Russia as yet. The great strides made towards civilisation by Russian cities of the first class leave little doubt, however, that a great development may be expected in this direction before many years are past

We were much interested in attending our first Russian dinner at a private house, that is a flat, for most people live in flats in St. Petersburg, except a few princes and other wealthy persons. During the warm weather most of the residents in St. Petersburg go out to the country, or at least to the Islands, a pretty suburb and favourite resort in the summer, which we have not space to describe. As our visit was paid early in September many people were still away, and those who had returned had not put up their winter curtains, etc., and so the house presented a somewhat dégagé appearance. During the first few weeks after the return of the families from the country, the temperature is in a sort of intermediate state; that is, neither hot enough for summer curtains nor cold enough to warrant the bringing out of winter hangings and other cosy articles. All this was explained to us by our friends, but the rooms in this intermediate condition were remarkably comfortable, and the omission referred to was not apparent to a visitor accustomed to a temperate climate. On entering the dining-room, instead of taking a seat at the table, the guests passed on to one end of the room where a table was placed containing the *zakuska* (*dinette*), consisting of relishes of various kinds—caviare, raw smoked goose, raw herrings, smoked salmon, tongue, cheese of various kinds, dried sturgeon, and other items of an unknown character, to which the natives are very partial. Bottles of liqueurs and wine were also there, the contents of which were drunk out of alarmingly large glasses shaped like a convolvulus, which are usually emptied with gusto by everybody. Quite a quarter of an hour is thus occupied in getting up an appetite, when the guests seat themselves at the dining table proper, and the dinner is served, with the wines to which we are accustomed at home. After dinner, tea is served up in tumblers, each being placed in silver or gold stands with a handle of the same metal, so that the contents may be drunk without burning the fingers. After this comes the dessert, consisting of a great variety of fruits, of which the Russians are wisely fond.

In hot weather—and it is at times very hot in Russia—iced soup is served called *batvénia*. This soup is very good, but the effect of the first spoonful upon the uninitiated, who may be expecting hot potage, is most comical, and reminded the writer of an incident which occurred at the banquet given to the Colonists in London at the time of the Colonial Exhibition, when ice pudding was served at the end of a sumptuous repast. Game of many kinds is procurable in abundance, the market in the winter presenting a wonderful spectacle, owing to the stiff and often grotesque appearance of the frozen birds. Game is best in the autumn, as the frost spoils the flavour. The iced soup, on the other hand, is most appetising and delicious, and might be introduced in the summer into England with advantage. The best of all the Russian game in our opinion is the *riabchik*, or hazel grouse. There are several sorts of fish to be had, one special kind, the sterlet, being very expensive, and costing about six shillings per person. This fish is very rich and tasty, but should be partaken of sparingly by all who have not the best of digestions and health. With the soup, and sometimes with the fish, is usually served a variety of patties made of shortbread, and containing sturgeon or some other game or meat, which invariably proved most appetising. A dish of which visitors should not fail to partake is the *pórosionok*, or sucking pig with horse radish sauce stuffed with buckwheat. Another purely Russian dish which proved very good was the *barány-boks-kashoi*, a roast leg of mutton also stuffed with buckwheat. The sweets are all excellent, being very popular with the Russians, who seem never tired of eating them. Several varieties of excellent wine are produced in the Crimea, the champagne, though rather sweet, being in other respects good, especially that of the Don; and another wine, a sort of light Burgundy called Kahétinskoe, should be asked for, as it is sound and pleasant.

The best hotels in St. Petersburg are the Hotel de l'Europe and the Hotel de France. The first is a magnificent building on the plan of the Metropole or Grand. Everything is very well done, and the charges on the whole are not unreasonable, but it is too large in our opinion for comfort. We much preferred the older and smaller Hotel de France, where everything is most comfortable and excellent, the proprietor and managers exerting themselves to the utmost to give satisfaction to everybody. The cooking at this hotel is especially good, and a dinner, consisting entirely of Russian dishes, of which we partook, was so well served as to be notable. We determined to have some sterlets, which were procured with great difficulty, and the manager was so pleased when he received them that he brought them in alive in a basket, out of which they jumped, to our amusement and to the astonishment of those who were in the restaurant, when he produced them for our inspection. They are about 18 to 20 inches in length with flat heads, and resemble a dagger in shape. The attendance at the hotels, and especially at the restaurants, is exceptionally good, the waiters at the latter being attired in bright-coloured or white silk blouses, or shirts with a band or belt, which costumes give a pretty effect to the salon. The Russian cooking is certainly preferable in our opinion to that of the French, and so far as the living is concerned there is little left to wish for; the Russian hotels outside St. Petersburg, however, leave much to be desired. At Moscow the hotels are not nearly as good as the two above referred to, and visitors would be well advised to choose the "Dussaux" rather than the larger and more pretentious establishment, the "Slavianski Bazaar."

The chief restaurant at St. Petersburg is the "Palkin Fraktir," which has a great reputation, which it is thoroughly entitled to. Visitors to Moscow would be well advised to take their meals at a restaurant rather than at hotels, the "Ermitage" and the "Bolshoi Moscovski" being the best. The former is said to have the best cuisine, and visitors will find that everything is not only well cooked but well served. These restaurants have large organs, played by clockwork, a list of music being presented to the guests, who can select their own pieces. It was strange to see two waiters winding up the organ and changing the key-boards, which are several feet in length and apparently very heavy. They present a striking appearance, as the shapes of all sorts of instruments are represented by the pipes, which are seen through the glass panels of the cases which contain the organ. The music is very good, and adds much to the comfort and enjoyment of the diners. There is a fixed tariff, but it is often difficult to order a dinner, or, at any rate, to change a dish during its course, as probably none of the waiters will understand any language except Russian, and the manager is not usually come-at-able when once the orders have been given.

(To be continued.)

